# QALYs: The Math Doesn’t Work

**DOI:** 10.36469/001c.83387

**Published:** 2023-07-27

**Authors:** Tia G. Sawhney, Angela Dobes, Sirimon O’Charoen

**Affiliations:** 1 Teus Health, LLC, Newark, New Jersey, USA; 2 School of Global Public Health, New York University, New York, USA; 3 Crohn’s and Colitis Foundation, New York, NY, USA; 4 Crohn’s & Colitis Foundation, New York, NY, USA

**Keywords:** quality-adjusted life-year, quality-of-life utility, cost-effectiveness, inflammatory bowel disease

## Abstract

The quality-adjusted life-year (QALY) is a metric widely used when assessing the cost-effectiveness of drugs and other health interventions. The assessments are used in the development of recommendations for pricing, formulary placement decisions, and health policy decisions. A new bill, H.R. 485, the Protecting Health Care for All Patients Act of 2023, was approved by the US House Energy and Commerce Health Subcommittee that will, if passed, end the practice of using QALYs in all federal programs.^1,2^

Proponents of the ban say that QALYs undervalue the positive effects of therapeutics on people with disabilities.^3^ We share their concerns. Furthermore, our review of the mathematical properties of QALYs, including an analysis of quality-of-life utility (QOL utility) data recently collected from patients with inflammatory bowel disease (IBD), has led us to conclude that QALYs are an inappropriate metric of drug and treatment cost-effectiveness for all people, both disabled and nondisabled, and should not be the basis for US healthcare policy decisions.

QALYs are time-based, health-related QOL utility measures. Health-related QOL utility measures purport to express the quality of a person’s life on a 0 to 1.00 scale, where 0 is death and 1.00 is perfect health.^1^ A QOL utility is converted to a QALY by multiplying the QOL utility by the number of years a person experiences that QOL.^4^

The mathematical appropriateness of using QOL utility measures in the denominator of cost-effectiveness assessments is predicated on 4 assumptions:

- Society has a common understanding of what constitutes perfect health.
- Imperfect health can be measured on a linear utility scale, relative to death and perfect health.
- Poor health is as likely to be measured as better health.
- Positive and negative health changes with the same absolute value, as measured by the utility scale, have equal societal value.

We examined these assumptions by reviewing QALY and QOL utility literature and by analyzing QOL utility data collected in late 2022 from 1038 people with IBD. The analysis of the IBD data allowed us to view the mathematical QOL utilities from the perspective of real-world patients whose treatment access may be curtailed if their drugs are deemed too expensive relative to QALY gains.

## Assumption 1: Definition of Perfect Health

Health is a multidimensional, highly personal experience. The World Health Organization defines perfect health as “a state of complete physical, mental and social well-being and not merely the absence of disease or infirmity,” a multidimensional definition that incorporates peace, security, and societal dangers.^5^ Central to the definition is “well-being,” a state that is dependent on personal perception.^6^

If we ask relatives, friends, and strangers how they measure their health and quality of life, their responses are likely to be as complex and unique as snowflakes. Yet, QOL measurement developers reduce quality-of-life measurements to a handful of simplistic questions. Furthermore, their question sets are inconsistent with each other and with common perceptions of health.

**About IBD**

Inflammatory bowel disease (IBD) is a term for two conditions, Crohn’s disease and ulcerative colitis, both characterized by chronic inflammation of the gastrointestinal tract. Common symptoms of IBD include persistent diarrhea, abdominal pain, rectal bleeding/bloody stools, weight loss, and fatigue. Prolonged inflammation results in permanent damage to the gastrointestinal tract.^7^ There are multiple treatment choices for IBD, including general and targeted immunomodulators (TIMs). Targeted immunomodulators, which include biologics, such as infliximab, and other targeted synthetic small molecules, such as JAK inhibitors, are quite expensive. Assessment of both the short-term and long-term cost-effectiveness of TIMs impacts IBD patient access to care.

For example, in 2019-2020, the Institute for Clinical and Economic Review (ICER) conducted an assessment of the TIMs for ulcerative colitis and determined that none of the TIMs were below ICER’s per-QALY cost-effectiveness price threshold.^8^ Assessments such as this can negatively impact getting the right treatment to the right patient at the right time. Patients may be denied drugs, may need to try and fail other drugs, and may be subjected to burdensome preauthorization procedures, all of which may lead to avoidable consequences, including hospitalization and surgery.

Consider the questions asked by the EQ-5D-5L QOL^9^ and PROMIS-Preference (PROPr) systems.^10^ (The EQ-5D-5L QOL is widely used in Europe and is the preferred instrument of the Institute for Clinical and Economic Review [ICER]. PROPr development was funded by the US National Institutes of Health^11^ and is gaining traction in the United States as an alternative QOL utility measure.)

According to EuroQOL, the developers of the EQ-5D-5L questions, a person is in perfect health if they report that, as of today, they:^12^

- Have no problems walking about
- Have no problems washing or dressing themselves
- Have no problems doing their usual activities (even if their usual activities are constrained)
- Have no pain or discomfort
- Are not anxious or depressed

In contrast, under the PROPr system, a person is in perfect health if they report that, over the last 7 days and in response to 29 questions, they have had no difficulties or negative experiences with physical function, anxiety, depression, fatigue, sleep disturbance, ability to participate in social roles and activities, and pain.^13^ Something as minor as a restless night of sleep, for any reason, means an imperfect PROPr score.

Different question sets to ascertain perfect health might be acceptable if the question sets identify approximately the same people as having 1.00 or near-1.00 health utilities. But they do not.

**Figure 1.**
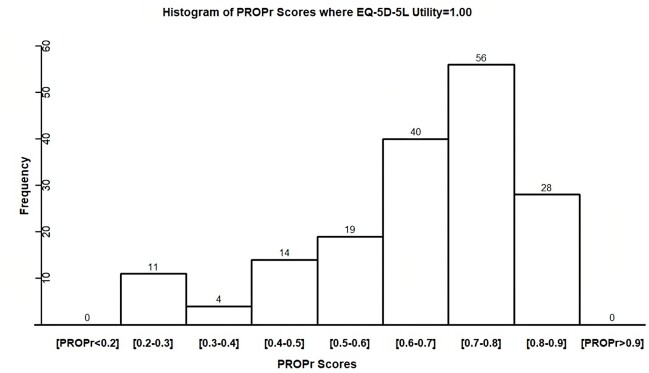
PROPr Utility Score for People in Perfect EQ-5D-5L Health Abbreviation: PROPr, PROMIS-Preference.

In late 2022 we asked 1038 IBD Partners survey participants to answer both EQ-5D-5L and PROMIS-29 questions.^14^ We then calculated EQ-5D-5L utility and PROPr scores. Per the EQ-5D-5L questions, 172 (16.6%) participants had perfect health (utility 1.00), which seems unlikely for a population with IBD, a chronic condition that impacts a person’s quality of life. None of the 172 “perfect health” participants per EQ-5D-5L, however, had perfect PROPr health; none even had a PROPr utility score above 0.90. The mean PROPr utility score for the people in perfect EQ-5D-5L health was 0.70 and 29 of the 172 participants (40%) had a PROPr score of 0.50 or less (**Figure 1**).

Our results are not anomalies. Vast intersystem differences in the identification of perfect health have been previously reported, including between the 3L and 5L versions of EuroQOL’s EQ-5D.^15^

## Assumption 2: Linear Values for Imperfect Health

Once there is agreement upon 1.00 health, all other health states need to be expressed on a linear 0 to 1.00 scale. “Linear” means that the health value of moving a fixed increment along the scale has the same value, regardless of where one starts on the scale. For example, if someone’s health declines from 1.00 to 0.90, that loss has the same value as the loss another person experiences when they move from 0.30 to 0.20.

Economists attempt to achieve utility scores linearity by asking people to make theoretical trade-offs between health states and then creating utility scoring formulas that theoretically produce linear relationships at the population level. The problem with theoretical trade-offs is that they are abstract, unconstrained by the real world. There is little *a priori* reason to believe that a collection of theoretical trade-off questions, posed to a general population, can result in an equation that scales the value of imperfect health into consistent increments of perfect health.

If utility measurements had different definitions of perfect health, but were otherwise linear, then the best line fit between the utility measurements of competing utility systems would be linear.^16^ The fit lines, however, are inevitably curved and quite imperfect.

**Figure 2.**
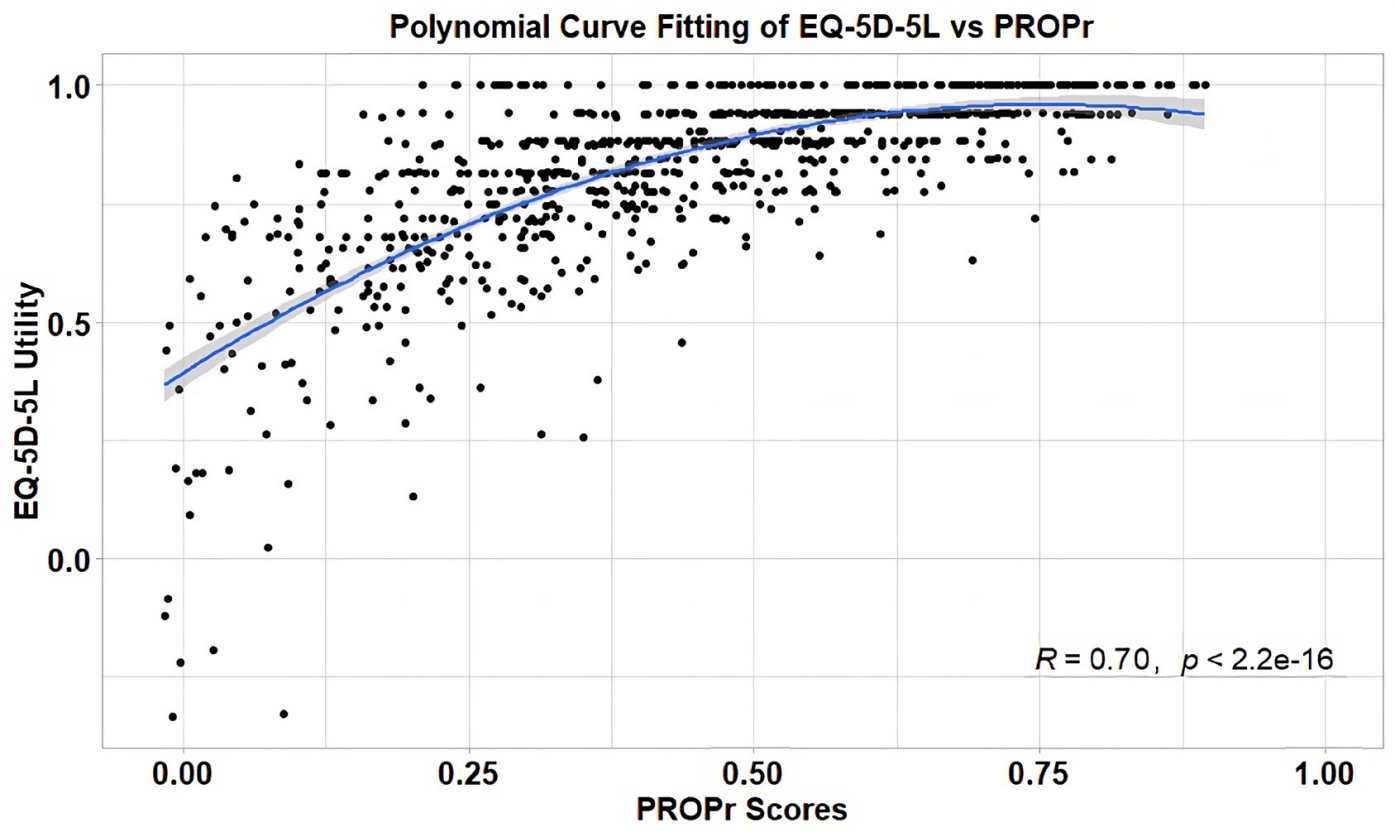
Fit Between PROPr Scores and EQ-5D-5L Utility Scores Abbreviation: PROPr, PROMIS-Preference.

**Figure 2** shows the fit between the PROPr scores and the EQ-5D-5L utility scores for our IBD survey participants.

## Assumption 3: Equal Measurement Likelihoods

QOL measurements rely on self-reported assessments of the patient’s recent health status (1 day for EQ-5D and 7 days for PROPr). Intuitively, we expect that people having a bad day or week are less likely to report their status than those having a good day or week. For example, people in the hospital or spending their day on a toilet and in severe pain are most often not answering surveys – they will want to skip the report or delay it until they feel better. The health impact of disease flares, which are common in IBD, is therefore likely underreported.

## Assumption 4: Offsetting Positive and Negative Health Changes

Our final required assumption is that positive and negative QOL utility changes with the same absolute value offset each other. This assumption is particularly discordant with societal values. Suppose a person with a significant disability is assigned a QOL utility of 0.30 and a person with a less-disabling chronic disease is assigned a QOL utility of 0.70. If QOL utilities are going to be the measurement we use to select between health policies, then the societal value of saving the disabled person’s life (avoiding a 0.30 loss) needs to have the same value as curing the other person’s chronic disease (gaining 0.30). Yet modern societies invest much more in preventing imminent death than in marginal QOL improvements. Furthermore, people with disabilities assert (rightfully) that their lives are not less worthy of saving than the lives of people with lesser impairments and higher QOL scores.

## Impact

As supported by our citations, none of our observations are new to economists. What’s surprising, however, is how often the failure of QALYs and QOL utilities to satisfy predicative assumptions is glossed over in the QALY and QOL utility literature with jargon and statistical modeling. While, as shown in **Figure 2**, there is clearly a correlation relationship between PROPr scores and EQ-5D-5L utilities that results in a relatively strong (0.49) *R*^2^ value, the correlation does not mean that either measurement is appropriate for use in healthcare policy decisions.

Because QOL utilities are expressed as values between 0 and 1.00, it is easy to dismiss the impact of a small absolute difference in a QOL utility or QOL utility increment value. Suppose different QOL utility systems estimate the impact of a health status change resulting from a drug treatment as 0.05 QALYs vs 0.15 QALYs – a “small” difference of 0.10. When these estimates are compared with treatment costs, the treatment is 3 times as expensive per incremental QALY via the first estimate. The estimation difference may be the difference between the treatment being recommended or not recommended.

## DISCUSSION

The passing of the 21st Century Cures Act ignited a transformational shift toward including the voice of the patient and their caregivers when assessing the value of innovative products and healthcare delivery.^17^ While progress has been made to incorporate the patient voice into research and care, patient-centered value remains not only an emerging concept but a concept that is not recognized in current health-related QOL utility measures.

When the first version of the EQ-5D instrument was developed in 1990, the researchers who developed it recognized the limitations of generalizing health into only 5 aspects (or “dimensions”). They declared that the instrument “is intended to complement other QOL measures and to facilitate the collection of a common data set for reference purposes.”^18^ It is now, however, being relied on as a primary, unitary measure of healthcare effectiveness.

It is critical for the researcher community to look beyond QOL utility and unitary measures in general and identify and test new methods for evaluating the value of healthcare interventions. The initiative by the National Health Council to develop Patient-Centered Core Impact Sets (PC-CIS) is an example of non-QOL-utility forward-thinking.^19^

PC-CIS is a patient-prioritized list of impacts a disease and/or its treatments have on a patient, their family, or caregivers. The impacts are intentionally broad and inclusive and include short-term and long-term health outcomes, and any other health-related implications. The standardized outcomes and implications can then be leveraged as the basis for downstream applications across the research, care, and access continuum, allowing for priorities to then be aligned across patients, caregivers, payers, providers, professionals and regulators, including product labeling language, clinical care guidelines and formulary placement decisions.

## CONCLUSION

We are not economists. We are, however, mathematically trained. We understand the convenience and appeal of a unitary measurement that can be used to evaluate all health interventions. But, regardless of convenience, a unitary measure must have consistent, linear, and symmetrical properties and be aligned with societal values. QALYs do not have such properties and should not be used for health decisions.

A metaphor affirms our conclusion. Suppose we were to measure the cost of a construction project in dollars ($) but not specify whether the dollars were US, Canadian, Singaporean, Australian dollars, or an unspecified cybercurrency that utilizes the $ sign. In addition, suppose that $20 was not necessarily 1/5 of $100, that $20 taken from one part of a project did not have the same value as $20 added to another part of the same project, and that negative budget variances may not be reported. Presumably none of us would be willing to make cost-based project decisions.

Neither the federal government, nor anyone else, should make healthcare policy decisions—decisions that impact people’s lives—based on unreliable, capricious QALYs and QOL utilities.

### Author Contributions

T.G.S. performed the data analysis and prepared the first draft of this article.

### Disclosures

A.D. and S.O. are employed by the Crohn’s & Colitis Foundation. T.G.S. has assisted several clients, including the Crohn’s & Colitis Foundation, with reviews of cost-effectiveness assessment and the use of QALYs and QOL utilities.

### Ethical Review

IBD Partners survey participants gave their consent for the research use of their survey responses. The University of North Carolina provided IRB approval for the collection of the survey data and sharing of data with the Crohn’s & Colitis Foundation. The Crohn’s & Colitis Foundation has an IRB waiver to use de-identified data to conduct this analysis.

## Footnotes

1 1.00 is alternatively described as “full health,” an equally nebulous concept. See, Aghdaee M, et al. Mapping the Patient-Reported Outcomes Measurement Information System (PROMIS-29) to EQ-5D-5L. *Pharmacoeconomics*. 2023;41(2):187-198.
